# High-resolution noncontact AFM and Kelvin probe force microscopy investigations of self-assembled photovoltaic donor–acceptor dyads

**DOI:** 10.3762/bjnano.7.71

**Published:** 2016-06-03

**Authors:** Benjamin Grévin, Pierre-Olivier Schwartz, Laure Biniek, Martin Brinkmann, Nicolas Leclerc, Elena Zaborova, Stéphane Méry

**Affiliations:** 1 University Grenoble Alpes, INAC-SPrAM, 38000 Grenoble, France; 2 CNRS Alpes, INAC-SPrAM, 38000 Grenoble, France; 3 CEA, INAC-SPrAM, 38000 Grenoble, France; 4 Institut de Physique et de Chimie des Matériaux de Strasbourg, Université de Strasbourg, CNRS UMR 7504, 23 rue du Loess, BP 43, 67034 Strasbourg Cedex 2, France; 5 Institut für Organische Chemie II und Neue Materialien, Ulm Universität, Albert-Einstein-Allee 11, Ulm, Germany; 6 Institut Charles Sadron, CNRS, Université de Strasbourg, 23 rue du Loess, BP 84047, 67034 Strasbourg Cedex 2, France,; 7 Institut de Chimie et Procédés pour l’Energie, l’Environnement et la santé (ICPEES), Université de Strasbourg, CNRS UMR 7515, ECPM, 25 rue Becquerel, 67087 Strasbourg Cedex 2, France

**Keywords:** donor–acceptor co-oligomers, donor–acceptor lamellae, donor–acceptor-ordered bulk heterojunction, Kelvin probe force microscopy (KPFM), noncontact atomic force microscopy (nc-AFM), organic photovoltaics, surface photo-voltage (SPV)

## Abstract

Self-assembled donor–acceptor dyads are used as model nanostructured heterojunctions for local investigations by noncontact atomic force microscopy (nc-AFM) and Kelvin probe force microscopy (KPFM). With the aim to probe the photo-induced charge carrier generation, thin films deposited on transparent indium tin oxide substrates are investigated in dark conditions and upon illumination. The topographic and contact potential difference (CPD) images taken under dark conditions are analysed in view of the results of complementary transmission electron microscopy (TEM) experiments. After in situ annealing, it is shown that the dyads with longer donor blocks essentially lead to standing acceptor–donor lamellae, where the acceptor and donor groups are π-stacked in an edge-on configuration. The existence of strong CPD and surface photo-voltage (SPV) contrasts shows that structural variations occur within the bulk of the edge-on stacks. SPV images with a very high lateral resolution are achieved, which allows for the resolution of local photo-charging contrasts at the scale of single edge-on lamella. This work paves the way for local investigations of the optoelectronic properties of donor–acceptor supramolecular architectures down to the elementary building block level.

## Introduction

Nowadays, with power conversion efficiency records of over 10% [1–2], solution-processed organic solar cells are regarded as a promising alternative to conventional silicon-based photovoltaic devices. Bulk heterojunction (BHJ) organic solar cells rely on blends of electron-donor (D) and electron-acceptor (A) materials, arranged in interpenetrated networks at the 10 nm scale to efficiently separate the excitons into free charges at the D–A interface. In the past decade, several studies demonstrated that Kelvin probe force microscopy (KPFM) can be powerfully combined with atomic force microscopy (AFM) to simultaneously probe the nanostructure and the optoelectronic properties of organic and hybrid, photoactive thin films and devices [3–9]. Particularly, the local surface photo-voltage (SPV) of organic blends [3–4,6–7] can be mapped in KPFM by analysing the surface potential (or contact potential difference, CPD) shift upon illumination. Nevertheless, analysing the SPV contrasts in relation with the nanostructure remains a challenge due to the complex morphology of nano-phase segregated D–A blends. Achieving a clear identification of the D–A interfaces in optimized, nano-phase segregated blends (i.e., at the 10nm scale) using KPFM continues to be a challenge. Moreover, recent studies have shown that pure donor and acceptor domains may often coexist with intermixed or co-crystallized phases [10–11]. In such structures, the donor and acceptor can be intimately mixed at the sub-10 nm scale. To establish the resolution limits of SPV imaging by KPFM, there is now a crucial need to investigate model D–A interfaces with better defined structural and electronic properties.

In that context, several groups have used the “microphase separation ability” of D–A block copolymers [12] or oligomers to elaborate well-defined, nanostructured D–A interfaces. Most of these supramolecular architectures rely on the use of fullerenes [13–15] or perylene-3,4,9,10-tetracarboxylic acid diimide (PDI) [16–20] as the acceptor components. However, to date, very few KPFM studies have focused on such self-assembled D–A networks [20].

**Figure 1:**
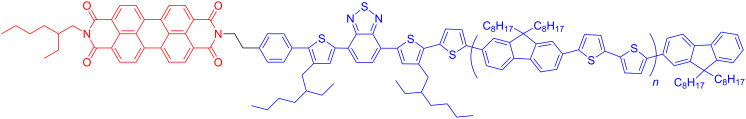
Molecular structures of the investigated A–D*_n_* co-oligomers (in this study *n* = 1 or *n* = 3).

In this study, we investigate a new class of PDI-based acceptor–donor block co-oligomers [19,21]. More precisely, the acceptor block (A) is PDI, whereas the donor block (D) is made of a combination of thiophene, ﬂuorene, and 2,1,3-benzothiadiazole derivatives (Figure 1). The donor length can be varied by repeating the number, *n*, of bithiophene-dioctylfluorene segments.

**Figure 2:**
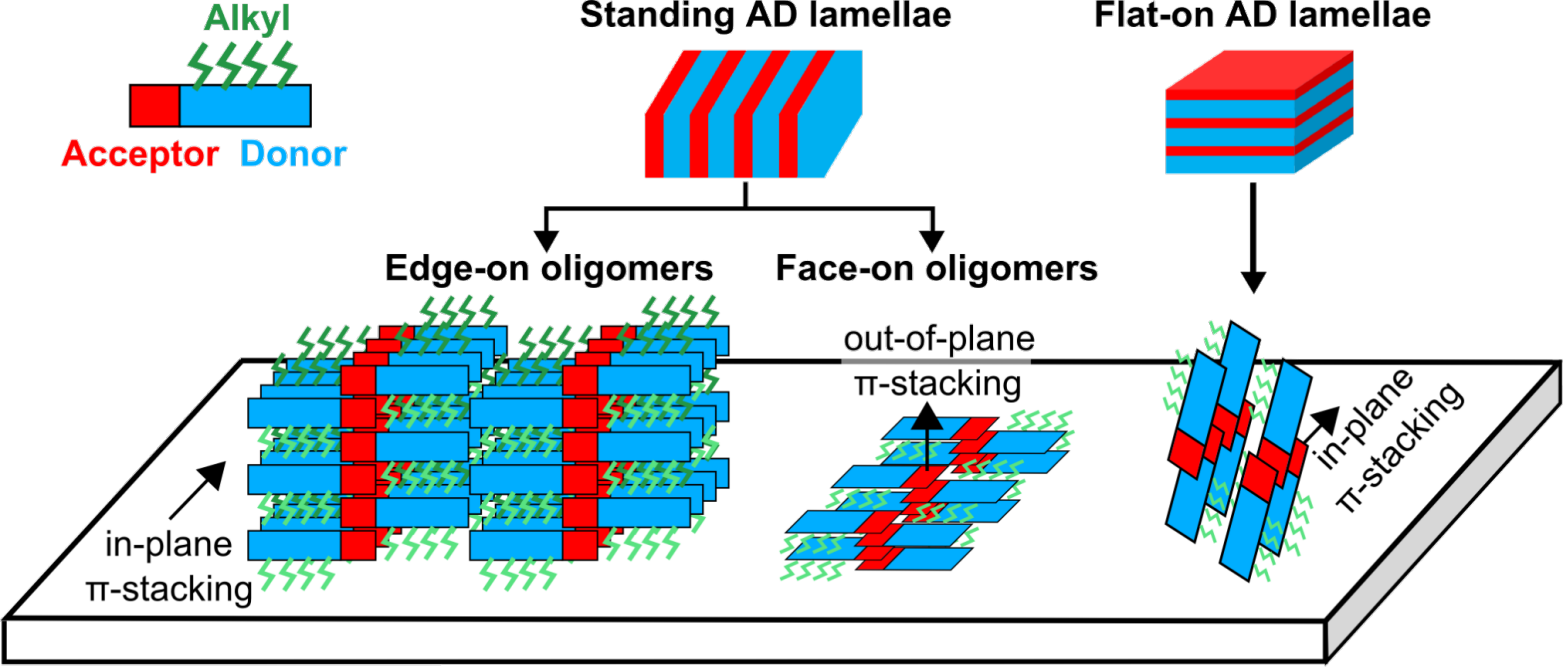
Schematic view of the self-assembled acceptor–donor (A–D) lamellar structures and the observed orientations on the substrate. In the present study, A–D films are composed of mostly standing lamellae with co-oligomers oriented in an edge-on configuration. In these domains, the molecules are π-stacked with a strong overlap of both the PDI and donor segments, and the π-stacking direction is parallel to the plane of the substrate (for more details see [19]). A minority of face-on co-oligomer molecules are also observed in standing A–D lamellae. Flat-on A–D lamellae are formed by co-oligomers oriented normal to the film surface.

Recent transmission electron microscopy (TEM) and X-ray diffraction (XRD) studies [19,21] have shown that these co-oligomers can form lamellar mesophases on various substrates (e.g., glass, SiO_2_, indium tin oxide, oriented poly(tetrafluoroethylene)). The D–A co-oligomers self-assemble in a zipper-like structure such that the donor groups and the PDI units form distinct D and A lamellae. In thin films, both standing and flat-on lamellae were evidenced as illustrated in the schematic of Figure 2.

For standing lamellae, the long axis of the molecules lies in the plane of the substrate whereas for the flat-on lamellae, the molecules are oriented approximately normal to the film plane. The ratio between standing and flat-on lamellae is a function of the donor block length as well as the processing conditions. Flat-on lamellae were obtained on oriented substrates of poly(tetrafluoroethylene) (PTFE) [21], while the films deposited on glass, SiO_2_ or indium tin oxide are mainly composed of standing lamellae. In these standing lamellae, two molecular orientations of the D–A oligomers with respect to the substrate are evidenced. They correspond to (i) edge-on co-oligomer orientation (π-stacking direction in the substrate plane) and (ii) face-on co-oligomer orientation with the π-stacking direction normal to the substrate. Both these molecular orientations coexist in the standing lamellae but with a majority of edge-on co-oligomers.

In this work, two samples with *n* = 1 (AD1) and *n* = 3 (AD3) were investigated by nc-AFM and KPFM in the form of thin films (a few tens of nm thick) deposited on functionalized indium tin oxide substrates. Most of the surface photo-voltage investigations were carried out on the AD3 sample, while the AD1 was mainly used in a first step to check the influence of the donor segment length on the film morphology. TEM measurements were carried out on other AD3 samples processed with similar conditions.

The manuscript will focus successively on i) the morphology characterization using AFM and TEM, ii) the analysis of the surface electrostatic contrast in dark conditions, and finally, iii) the surface photo-voltage characterization of the films under illumination.

## Methods

The nc-AFM experiments were performed in frequency modulation (FM) mode with an Omicron VT-AFM setup under UHV at room temperature. For each image, the frequency shift, Δ*f*, and vibration amplitude, *A*_Vib_, are indicated in the corresponding figure caption. Silicon cantilevers (SuperSharpSilicon, Nanosensors, n+-doped, stiffness 40 N/m, resonance frequency in the 280–300 kHz range) were Ar-sputtered in vacuum to remove the oxide layer and possible contaminants. KPFM measurements were performed in FM mode with the modulation bias *V*_AC_ (typically 1 V peak-to-peak at 900 Hz) and the compensation voltage *V*_DC_ applied to the cantilever (tip bias *V*_tip_ = *V*_DC_). In that configuration, the contact potential difference (CPD) is equal to −*V*_DC_. In this work, the potentiometric data are presented as compensation bias (*V*_tip_ = −CPD) images (also called CPD images, KPFM potential or surface potential images). Surface photo-voltage (SPV) images were calculated as the difference between the compensation bias images recorded under selective illumination and in dark conditions, SPV = *V*_tip_^illum^ − *V*_tip_^dark^. The SPV images were smoothed by applying a Gaussian filter (raw data display the same features but with a slightly higher noise level, see Figures S6, S7, and S8 in Supporting Information File 1).

The lateral lag (due to thermal drift and piezoelectric actuator creep) between the set of images used for the SPV calculation was corrected by using the lattice tool of the WsXM software [22] (see Figure S9 in Supporting Information File 1). After correcting the images, the residual lateral error in the alignment was estimated by comparing topographic cross-section profiles extracted with the multiple profile tool of WsXM (see Supporting Information File 1 for more details). The estimated lateral resolution is indicated for each SPV image in the corresponding figure caption.

Dyads thin films (50 ± 5 nm thick, determined from intermittent-contact AFM measurements, not shown) were deposited from toluene solutions (10 mg/mL) via spin-coating on indium–tin–oxide (ITO) functionalized with PEDOT:PSS (thickness 40 ± 5 nm). In this study, PEDOT:PSS was primarily used to reduce the roughness of the substrate. In situ sample annealing (60 min at 200 °C for AD1 and at 215 °C for AD3) has been performed at pressures of 10^−10^ mbar and under temperature control with an optical pyrometer. Similar results (not shown in this report) were obtained on thin films annealed at a slightly lower temperature (180 °C).

**Figure 3:**
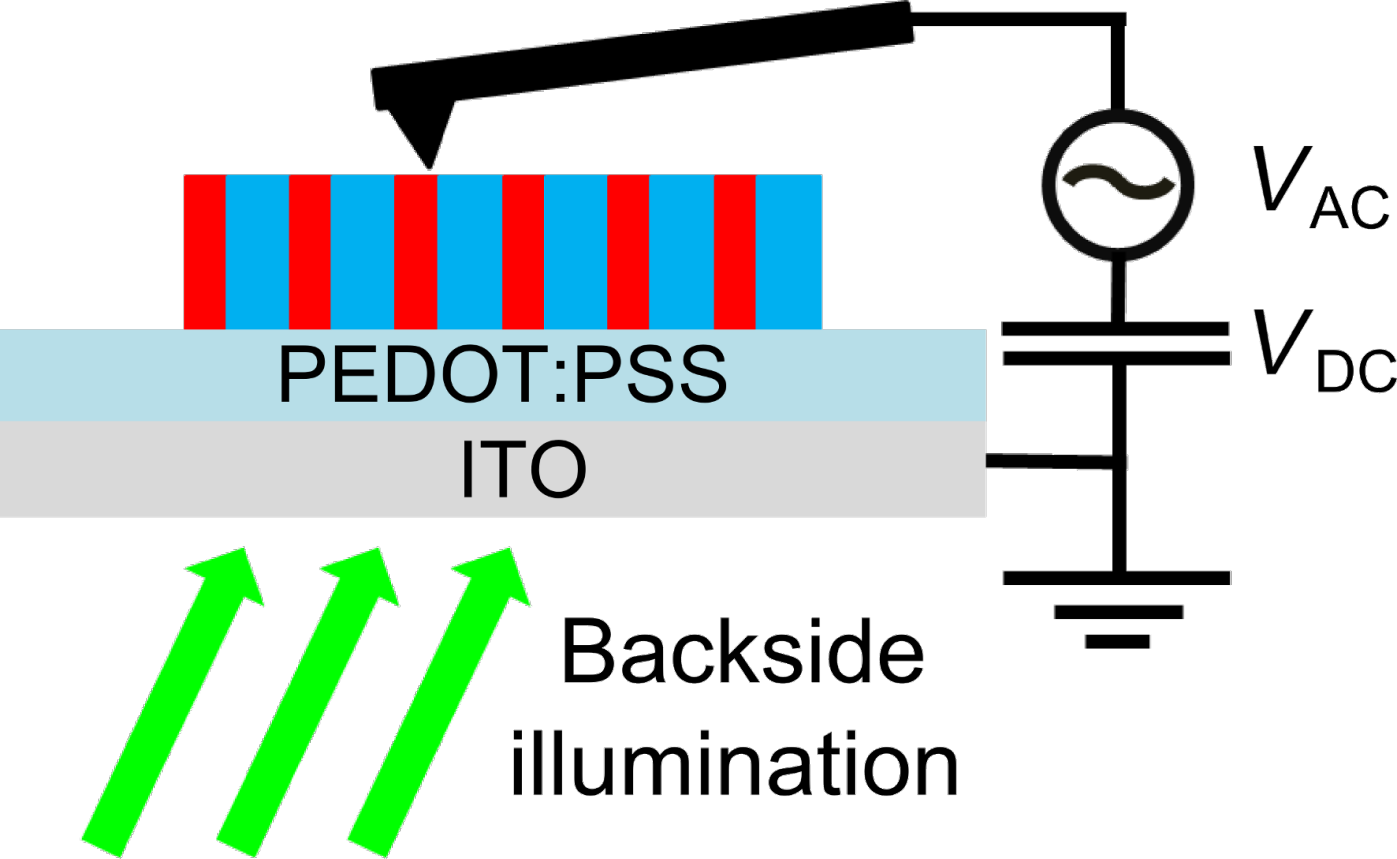
Geometry of the experimental setup for KPFM. The sample is illuminated in backside geometry. The modulation and compensation bias are applied to the tip, while the ITO substrate is grounded.

A triple laser source emitting at λ = 405 nm, 515 nm and 685 nm (spot beam diameter *≈*0.8 mm) with variable light intensity (OmicronLaser, Germany) was used for sample illumination (through an optical viewport of the UHV chamber). The sample was illuminated with a backside geometry (Figure 3) using specifically designed sample holders with on-board mirrors. The surface photo-voltage of the dyads was investigated as a function of the illumination wavelength, confirming the absence of any photo-voltage related to the silicon cantilever itself (see Figure S5 in Supporting Information File 1). In the following, all KPFM measurements under illumination were performed at a wavelength of 515 nm with an optical power of 15 mW.

For TEM investigations, the thin films were coated with a thin amorphous carbon film and removed from the glass substrate by floating on a diluted aqueous HF solution (10 wt %) and subsequent recovery on TEM copper grids. TEM was performed in bright field, high-resolution and diffraction modes using a CM12 Philips microscope equipped with a MVIII (Soft Imaging System) CCD camera. In order to avoid beam damage to the thin films, after focusing and correction of astigmatism, the electron beam was blanked with a shutter and a nearby area was selected to record the HRTEM image. Image treatment was performed by using the AnalySIS software (Soft Imaging System).

## Results and Discussion

### Surface morphology

**Figure 4:**
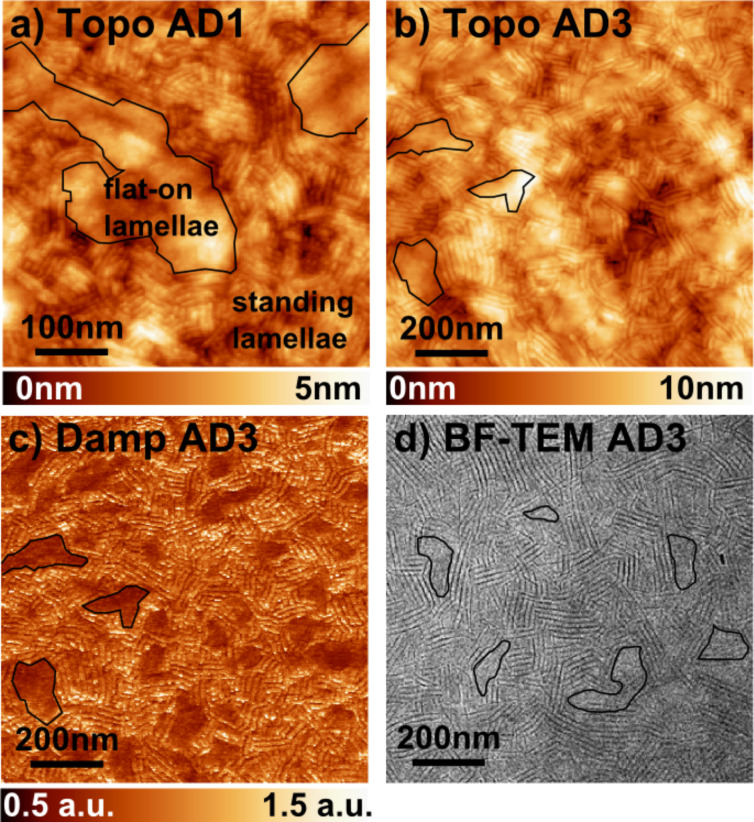
(a) nc-AFM topographic image (500 × 500 nm) of the AD1 film on ITO/PEDOT:PSS (Δ*f* = −55 Hz, *A*_Vib_ = 14 nm). (b,c) nc-AFM topographic (b) and damping (c) images (1000 × 1000 nm) of the AD3 film on ITO/PEDOT:PSS (Δ*f* = −20 Hz, *A*_Vib_ = 20 nm). (d) Bright field TEM image of an AD3 film. The area corresponding to some flat-on lamellae are highlighted by black contours in (a–d).

**Figure 5:**
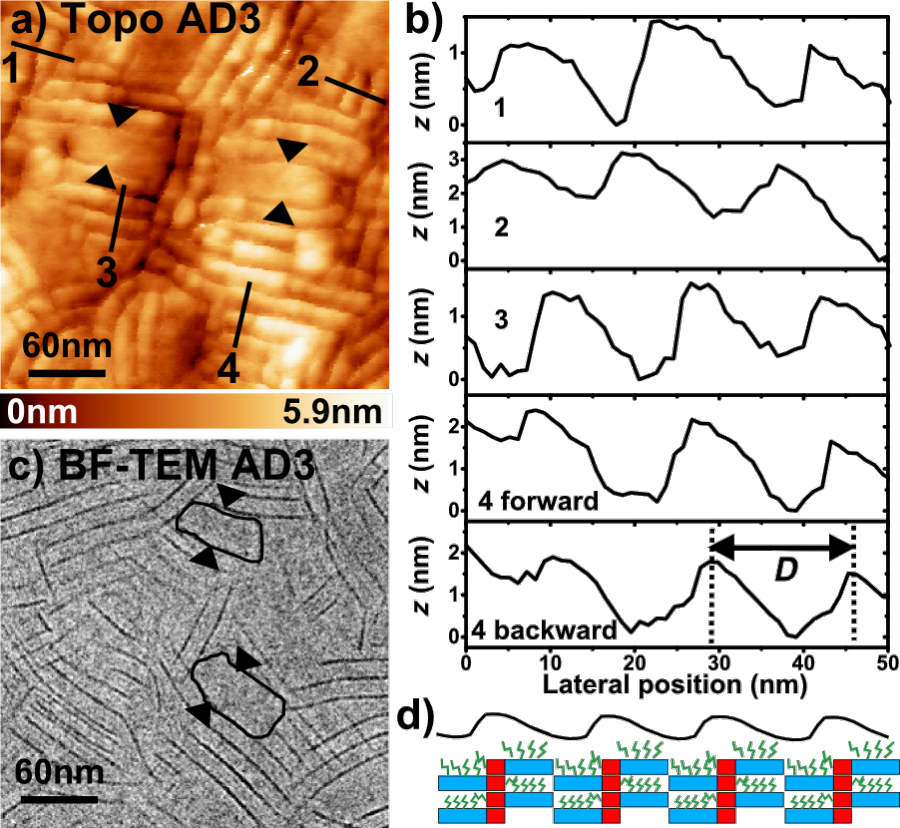
(a) 300 × 300 nm nc-AFM topographic image of the AD3 film (Δ*f* = −10 Hz, *A*_Vib_ = 20 nm). (b) Topographic profile corresponding to the path highlighted by the black line in (a).The two arrows pinpoint the location of a structural defect. (c) Bright field TEM image of an AD3 film showing identical defects. (d) Schematic side view of the edge-on lamellae, showing how the zipper-like organization results in the appearance of asymmetric AFM topographic profiles (black line).

Figure 4a,b shows the surface morphology of AD1 and AD3 films after in situ annealing. The topographic images reveal a lamellar self-organization with modulation periodicities (mean values deduced from FFT images, not shown) of ≈9.5 and 15 nm for AD1 and AD3, respectively. These values are fully consistent with the structural model proposed by Schwarz et al. [19] for standing A–D lamellae consisting of edge-on co-oligomers (Figure 2 and Figure 5d). We note that the higher level of damping recorded over the standing domains may be reasonably attributed to the influence of the lateral alkyl side groups, which point out of the surface in the case of the edge-on oligomers (see Figure 2).

It is also clear that the standing lamellae coexist with other domains (highlighted in Figure 4a–c) giving rise to a strong contrast in the dissipation images, as shown by Figure 4c. In these parts of the surface, the supramolecular packing cannot be directly resolved from the nc-AFM data. In turn, the similarity between the damping and TEM images is remarkable when comparing Figure 4c and Figure 4d. Actually, XRD and TEM investigations [19,21] have shown that these other domains consist of flat-on lamellae (see Figure 2) and that their proportion in the film is inversely proportional to the length of the donor blocks. This last result is consistent with the surface morphology probed by nc-AFM (compare Figure S1a and Figure S1b in Supporting Information File 1). In the case of the longer AD3 dyad, a statistical analysis performed on large-scale damping images (by using the “flooding” tool of the WSxM software [22], not shown) shows that less than 16% of the surface is apparently covered by flat-on domains. Thus, the AD3 films are mostly composed of standing lamellae, which can be used as model A–D networks for local surface photo-voltage investigations by KPFM. They provide a benchmark to check the ability to resolve the contributions to the SPV contrasts of donor and acceptor units.

Interestingly, the topographic profiles acquired over the standing lamellae (Figure 5a,b) reveal not only a periodic modulation, but also a characteristic asymmetry in the section profile as shown in Figure 5b. This phenomenon is observed both for the forward and backward scans, and is independent of the scanning direction relative to the lamellar in-plane orientation. Moreover, this has been observed by using different cantilevers (not shown). As depicted in Figure 5d, this asymmetry is consistent with the structural model [21] for the standing lamellae with edge-on co-oligomers. Indeed the zipper-like stacking results in a factory roof profile as observed by AFM. Besides, structural defects within the edge-on stacks are revealed in nc-AFM (Figure 5a) and TEM (Figure 5c) images, and appear as local doubling of the lamellar periodicity. They may exist either as small face-on domains or inclined lamellae.

### Electrostatic contrasts under dark conditions

**Figure 6:**
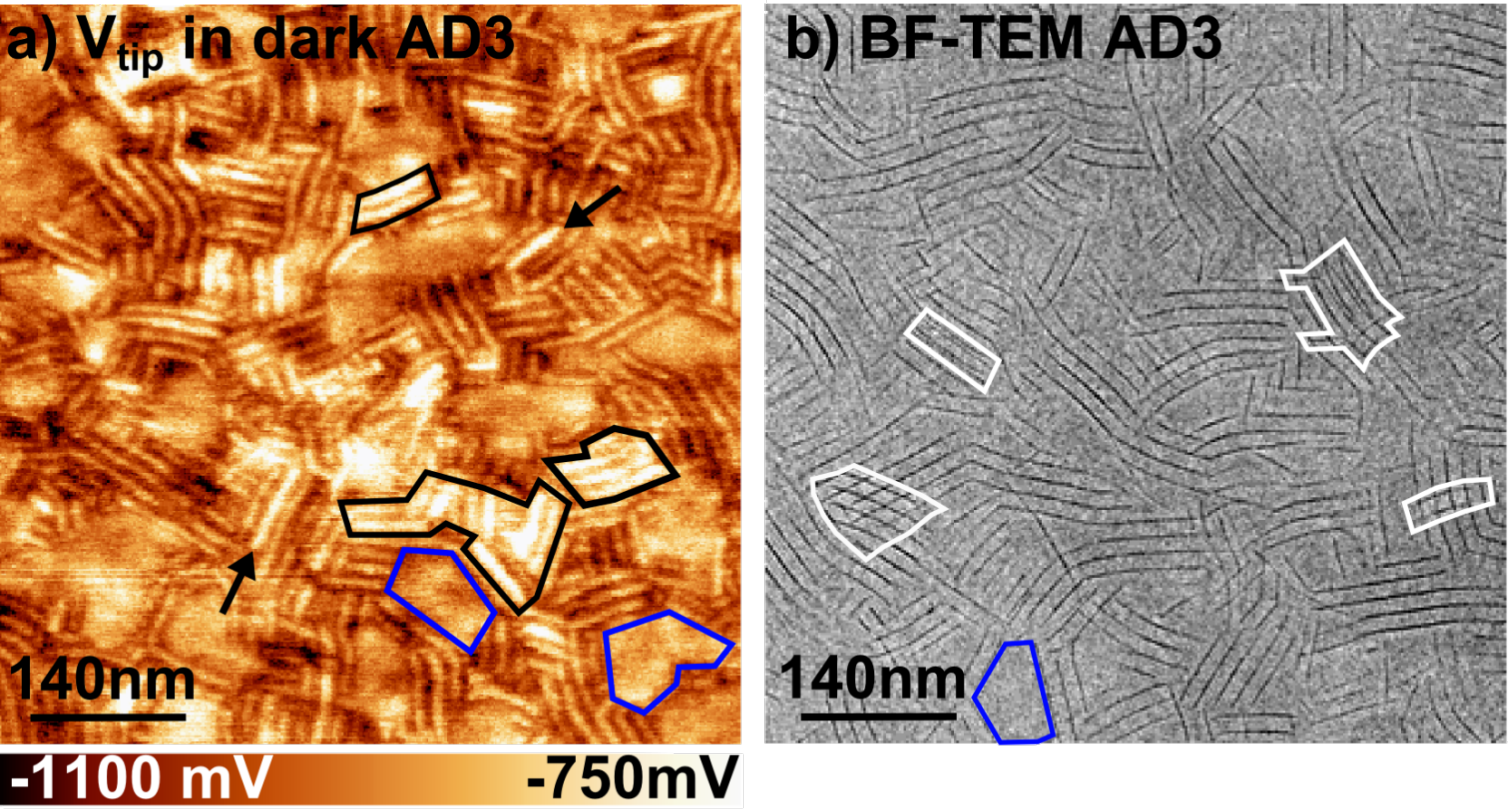
(a) KPFM potential image of the AD3 film recorded in dark (670 × 670 nm, Δ*f* = −20 Hz, *A*_Vib_ = 20 nm). (b) Bright field TEM image of another AD3 film at the same scale. In both images, flat-on lamellae are highlighted by blue contours. The edge-on lamellae with a high KPFM potential level and the cross-hatch patterns are highlighted by black and white contours in (a) and (b), respectively. Black arrows in (a) pinpoint individual lamellae with higher surface potentials.

Further insight into the structural organization can be achieved by analysing the contact potential images in dark conditions (Figure 6a). In principle, for these undoped oligomers, one expects no permanent charges under dark conditions, with the exception of molecular dipoles and interface dipoles at the dyad–substrate interface.

Firstly, we note the absence of any significant CPD contrast within the flat-on lamellae (blue contours in Figure 6), which is consistent because the tip probes the top surface consisting exclusively of donor groups. Besides, the mean potential level is nearly the same over all flat-on domains. This last point reveals the absence of structural variations within the bulk of the film. In such a case, the CPD originates only from the interface dipole at the recessed dyad–PEDOT:PSS interface.

In turn, the KPFM potential displays modulations over the edge-on domains, in correspondence with the topography and damping channels (see Figure S2 in Supporting Information File 1). Moreover, the KPFM potential image shows additional features not apparent in the other channels. These features correspond essentially to individual or a few A–D lamellae with strong potentiometric contrasts, mostly in the form of brighter stripes in the CPD images. The absence of equivalent features in topography and dissipation images (Figure S2 in Supporting Information File 1) proves the absence of artefacts at the origin of these CPD contrasts, which therefore originate from uneven charge distributions within the edge-on domains.

The most likely explanation is that structural variations occur below the surface of the standing lamellae. These defects impact the molecular dipoles at the surface of the film, due to the mutual polarization effects and wave function overlap with the recessed layers.

Especially, the top layers may cover recessed edge-on stacks displaying a different π-stacking direction, or eventually sub-surface flat-on domains. The first hypothesis is particularly supported by the existence of cross-hatch patterns in the TEM images (Figure 6b), clearly revealing that lamellae with different π-stacking directions overlap in some parts of the film. The TEM bright field images correspond to 2D projections of a 3D film morphology. Hence, cross-hatched patterns indicate that two layers of standing lamellae with two different azimuthal orientations are superposed (Figure S3 in Supporting Information File 1).

Here, we stress that in many cases, the size of the “bright CPD patches” is compatible with that found for the cross-hatched areas (compare Figure 6a and Figure 6b). With regards to the contrasts displayed at the scale of individual lamellae (highlighted by black arrows in Figure 6a), one could consider the existence of standing lamellae with face-on co-oligomer orientation. However, this hypothesis is excluded by the analysis of topographic profiles extracted from high-resolution images (see Figure S2 in Supporting Information File 1). Hence, these features most probably reflect underlying structural defects in the bulk of the standing lamellae.

All in all, these results support a picture in which flat-on domains percolate through the whole film thickness without significant structural changes, while edge-on domains present structural variations in the bulk of the sample.

### Photo-voltage imaging

**Figure 7:**
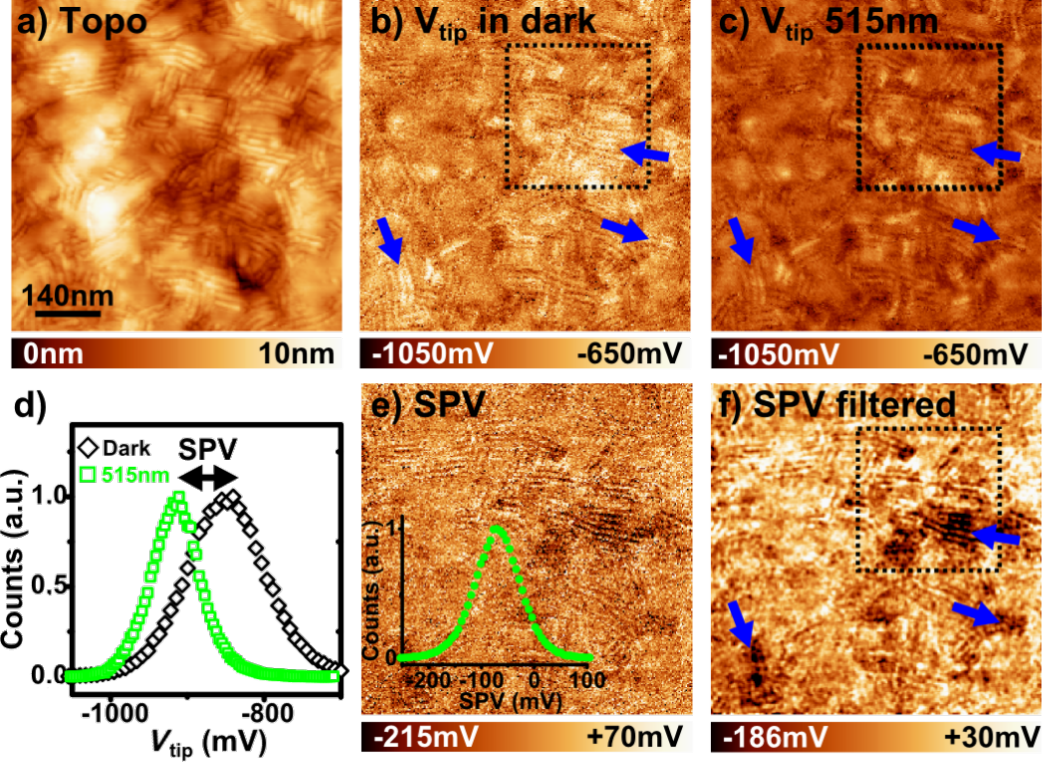
(a–c) nc-AFM/KPFM images (712 × 712 nm) of the AD3 film (Δ*f* = −10 Hz, *A*_Vib_ = 20 nm) recorded in dark conditions (a,b) and under illumination (c). (d) Histograms of the surface potential values calculated from (b) and (c). (e) SPV image calculated as the difference between (c) and (b). Lateral resolution: *≈*5 nm (see Figure S6 in Supporting Information File 1). The corresponding SPV histogram is given in the inset. (f) SPV image filtered with a Gaussian smoothing function. The blue arrows in (b,c,f) indicate some areas where the potential collected under dark conditions is higher and the SPV is more negative. The black dotted squares indicate the location corresponding to the high-resolution images (shown later in Figure 9a,b).

In the following, we focus on the analysis of the surface photo-voltage (SPV) images acquired on the AD3 sample. At the mesoscopic scale (Figure 7), it can be clearly seen that the surface potential shifts downwards upon illumination, resulting in a negative surface photo-voltage. The potential shift is completely reversible (Figure S4 in Supporting Information File 1), revealing the absence of permanent charge trapping effects in these samples.

The SPV displays a logarithmic dependence as a function of the illumination intensity [4], which is related to the electron–hole recombination kinetics [23]. More precisely, the slope of the SPV as a function of the natural logarithm of the intensity (see Figure S4 in Supporting Information File 1) is equal to *k*_B_*T*/*e* = 25 mV, which shows that the recombination is bimolecular [23]. Besides, the photo-voltage response as a function of the illumination wavelength is consistent with the absorption spectrum of the dyads (see Figure S5 in Supporting Information File 1).

**Figure 8:**
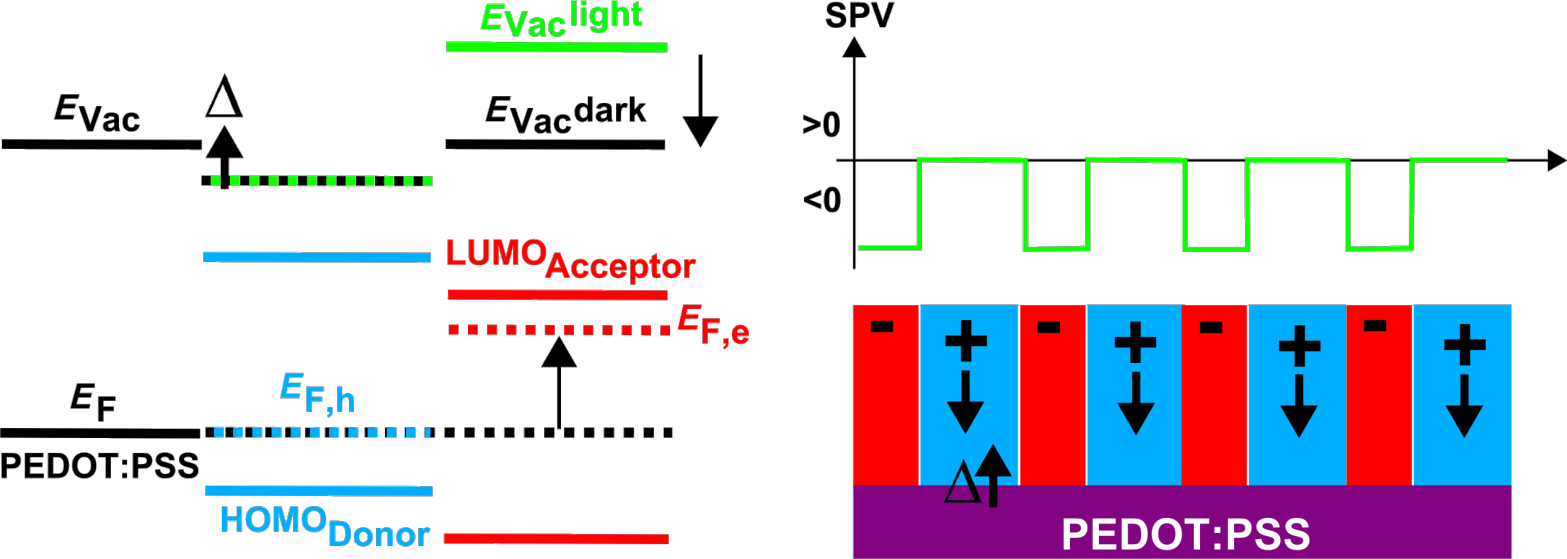
Schematic representation of an idealized D–A network and its band alignment with respect to the substrate. Here, we assume a Fermi level pinning of the donor blocks after charge transfer to the substrate (resulting in the existence of an interface dipole, Δ), and a flat band alignment (in dark) of the acceptor segments. Under illumination, the local vacuum level (*E*_Vac_) remains constant over the donor segments while it is shifted upward over the acceptor segments, resulting in a negative shift of the surface potential in average. *E*_F_: Fermi level. *E*_F,h_ (*E*_F,e_): quasi-Fermi level of holes (electrons).

In organic donor–acceptor solar cells, the SPV measures the splitting of the quasi-Fermi levels of the holes and electrons under illumination across the donor–acceptor interface [8]. In operating devices, the quasi-Fermi levels of the holes and electrons are nearly aligned with the Fermi levels of the anode and cathode, respectively. The SPV matches the open circuit voltage and is negative when the anode is grounded. Here, the situation is more complicated. The ITO substrate is also grounded, but in the absence of a top metallic cathode, both donor and acceptor units can contribute to the local SPV measured at the surface of the film. However, it can be simply shown that the average SPV remains negative. In order to prove this, we consider the case of the idealized D–A network depicted in Figure 8. In the dark condition state, the Fermi level of the donor units is pinned by the substrate, while the acceptor blocks display a flat band alignment. Under illumination, the holes quasi-Fermi level remains aligned with the Fermi level of the grounded substrate, while the quasi-Fermi level of the electrons is located near the lowest unoccupied orbital level (LUMO) of the acceptor units. As a consequence, the local vacuum level remains constant over the donor units, and is shifted upward over the acceptor segments, resulting in a global downward shift of the surface potential (i.e., a negative SPV).

Now, the question that presents itself is whether this argument still holds in the case of more complex sample morphologies. In our case, it has been previously shown that the AD3 film consists mainly of standing lamellae constituted by edge-on molecules. Then, bi-continuous A–D networks will percolate through a large fraction of the whole film thickness, resulting in a negative SPV (on average).

Moreover, the SPV image displays well-marked contrast over the edge-on stacks (see Figure 7f), most of time in the form of dark patches. Actually, these local SPV minima occur over the lamellae appearing as bright stripes in the CPD images collected under dark conditions (see Figure 7 and Figure S7 in Supporting Information File 1). Again, we stress that the size of these dark SPV patches is compatible with that found for the cross-hatched areas in TEM images. Therefore, these last observations reinforce the hypothesis of structural variations within the bulk of the standing lamellae.

Here, it is mandatory to check that the local photo-potential contrasts have a physical origin and do not result from a misalignment of the set of source images used for the SPV calculation. In the case of the image displayed in Figure 7f, the analysis of topographic profiles recorded simultaneously with the KPFM data in dark and under illumination (Figure S6, Supporting Information File 1) shows that the lateral misalignment is at a maximum of 5 nm. This value falls well below the lateral extension of the area displaying a lower photo-voltage (highlighted by blue arrows in Figure 7), which is on the order of 100 nm. Therefore, the lateral resolution is sufficient to establish that the SPV contrasts are related to heterogeneities in the photo-carrier distribution under illumination.

**Figure 9:**
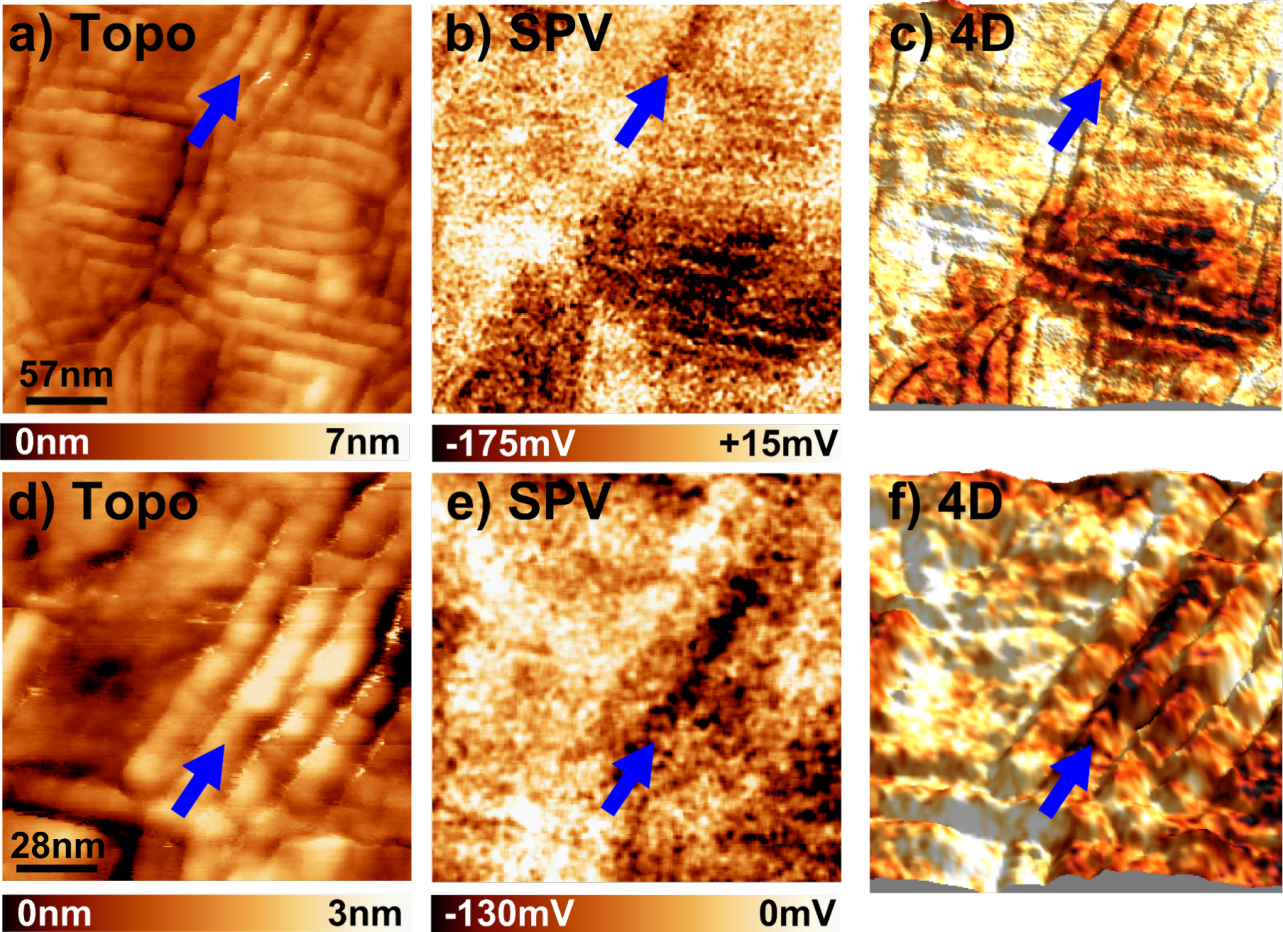
(a) 286 × 286 nm nc-AFM topographic image of the AD3 film. (b) SPV image calculated as the difference between the KPFM images recorded under illumination and in dark (see Figure S7 in Supporting Information File 1). Lateral resolution: *≈*2 nm. (c) 4D textured image (topography + SPV) obtained by applying the color code used for the photo-potential values of the SPV image onto a 3D representation of the topography. (d) High-resolution 145 × 145 nm nc-AFM topographic image. (e) Calculated SPV image. Lateral resolution: *≈*1 nm (see Figure S8 in Supporting Information File 1). (f) 4D textured image. The blue arrows pinpoint a supramolecular stack displaying a lower (i.e., more negative) SPV than its neighbors.

Besides the bright and dark mesoscopic patches, high-resolution SPV images (Figure 9) also display dark stripes corresponding to individual A–D lamellae. The image analysis reveals that these local SPV contrasts are actually correlated with the supramolecular lattice. One lamella, displaying an SPV lower than its neighbour, is highlighted in Figure 9b,c and Figure 9e,f. For the latter data sets (Figure 9e,f), the lateral resolution falls (on average) below 1 nm (see Figure S8 in Supporting Information File 1), unambiguously demonstrating that the lower SPV is intrinsically related to the charging state of a single lamella.

Now we briefly discuss the origin of the observed SPV contrasts. As mentioned above, it is likely that the local SPV minima (in other words, the “dark” SPV patches) reflect the existence of recessed defects within the bulk of the film. Especially, stacking several layers of edge-on oligomers with different orientations should dramatically impact the electron delocalization through the bulk of the film by breaking the continuity of the subnetwork formed by the acceptor units. In turn, the hole transport will be less affected, due to the longer length of the donor segments. Then, disorder-induced electron localization could be the origin of the local negative SPV contrasts.

At this stage, we cannot definitely conclude this – particularly since the structural organization of the recessed layers remains partially unknown. Further experiments, such as mapping the morphology and SPV for films of different thicknesses (down to a few monolayers), will be needed to conclusively address this issue. Local measurements of the recombination rates [24] may also help to establish a more comprehensive picture of the local photo-transport mechanisms in these self-assembled D–A architectures.

A final topic we want to address is the ability to resolve the contributions to the SPV contrasts of donor and acceptor units within a single A–D lamella. Numeric zoom within high-resolution SPV images shows that the minima of the topography match approximatively with the maxima of the surface potential (Figure S10 in Supporting Information File 1), and vice versa. This contrast inversion seems, in a first approximation, consistent with the structural model envisioned for the edge-on lamellae. Indeed, if one takes into account the dynamic motion of the lateral alkyl side groups, the topographic maxima should be close to the location of the negatively charged PDI units. However, a direct visualisation of the donor and acceptor segments remains beyond the limits of resolution of our experiment. At room temperature, the thermal fluctuations of the alkyl segments prevent more precise resolution at the supramolecular scale. We anticipate that cryogenic nc-AFM/KPFM experiments [25] will help to reveal the exact nature of SPV contrasts at the submolecular scale in these self-assembled dyads.

## Conclusion

Self-assembled thin films of donor–acceptor dyads have been investigated by noncontact atomic force microscopy and Kelvin probe force microscopy. Consistent with the results of transmission electron microscopy, the nc-AFM images reveal that the dyads self-assemble primarily as edge-on lamellae, and to a lesser extent, as flat-on domains. The comparison with the TEM results suggests that structural variations within the bulk of the edge-on domains could be at the origin of the electrostatic contrasts probed by KPFM.

Specific features observed in KPFM and SPV images may to some extend be related to structural defects evidenced by TEM in the bulk of the films such as cross-hatched layers. SPV imaging reveals that structural defects in A–D co-oligomers affect the carrier photo-transport, and hence, the global photovoltaic properties of corresponding devices. Lastly, SPV contrasts were resolved at the scale of single edge-on lamellae, which is an important step towards local investigations of photovoltaic self-assembled donor–acceptor heterojunctions at the submolecular scale.

## Supporting Information

Comparison of nc-AFM topographic images of the AD1 and AD3 films. nc-AFM/KPFM images (topography, damping, CPD) of the AD3 film recorded in dark conditions. 3D representation of two lamellae with different in-plane π-stacking directions. Surface photo-voltage as a function of the illumination intensity. KPFM potential images recorded in dark and under illumination at 685 nm, 515 nm and 405 nm. Estimation of the SPV lateral resolution (3 series of topographic and KPFM potential images recorded in dark and under illumination). Correction procedure used for the SPV image calculation. High-resolution topographic and SPV cross sections over an edge-on lamellae.

File 1 Additional experimental results.
