# Supplementary material for: High-resolution noncontact AFM and Kelvin probe force microscopy investigations of self-assembled photovoltaic donor–acceptor dyads
Source: Beilstein J Nanotechnol. 2016 Jun 3;7:799–808. doi: 10.3762/bjnano.7.71 (PMC4902083; doi:10.3762/bjnano.7.71)
Supplement: File 1 — Additional experimental results. [file Beilstein_J_Nanotechnol-07-799-s001.pdf]

Supporting Information

for

High-resolution noncontact AFM and Kelvin probe force microscopy investigations of self-assembled photovoltaic donor–acceptor dyads

Benjamin Grévin*[1,2,3], Pierre-Olivier Schwartz[4,5], Laure Biniek[6], Martin Brinkmann[6], Nicolas Leclerc[7], Elena Zaborova[7], and Stéphane Méry*[4]

Address: [1]University Grenoble Alpes, INAC-SPrAM, 38000 Grenoble, France, [2]CNRS Alpes, INAC-SPrAM, 38000 Grenoble, France, [3]CEA, INAC-SPrAM, 38000 Grenoble, France, [4]Institut de Physique et de Chimie des Matériaux de Strasbourg, Université de Strasbourg, CNRS UMR 7504, 23 rue du Loess, BP 43, 67034 Strasbourg Cedex 2, France, [5]Current address: Institut für Organische Chemie II und Neue Materialien, Ulm Universität, Albert-Einstein-Allee 11, Ulm, Germany, [6]Institut Charles Sadron, CNRS, Université de Strasbourg, 23 rue du Loess, BP 84047, 67034 Strasbourg Cedex 2, France, and [7]Institut de Chimie et Procédés pour l'Energie, l'Environnement et la santé (ICPEES), Université de Strasbourg, CNRS UMR 7515, ECPM, 25 rue Becquerel, 67087 Strasbourg Cedex 2, France

Email: Benjamin Grévin* - benjamin.grevin@cea.fr; Stéphane Méry* - stephane.mery@ipcms.unistra.fr

* Corresponding author

Additional Experimental Results

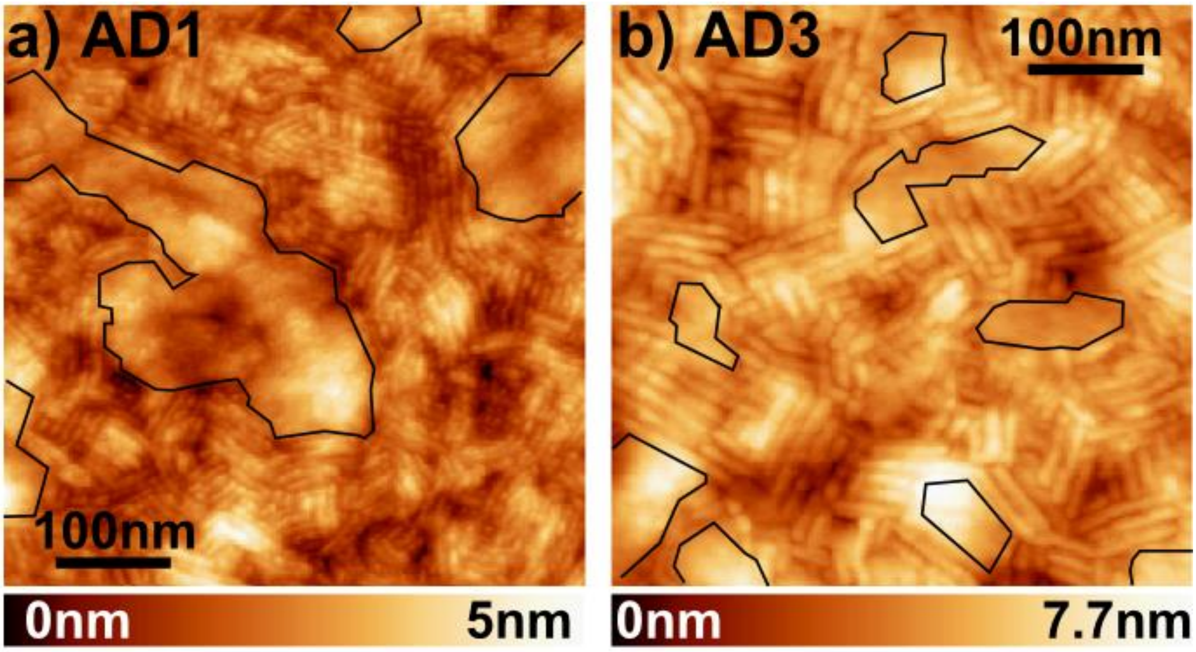

Figure S1: 500×500nm nc-AFM topographic images of the AD1 (a) and AD3 (b) films on ITO/PEDOT:PSS. The area corresponding to flat-on lamellae are highlighted by black contours in (a) and (b). At this scale the apparent surface coverage by flat-on domains is estimated to *ca.* 25% and *ca.* 13% for AD1 and AD3, respectively.

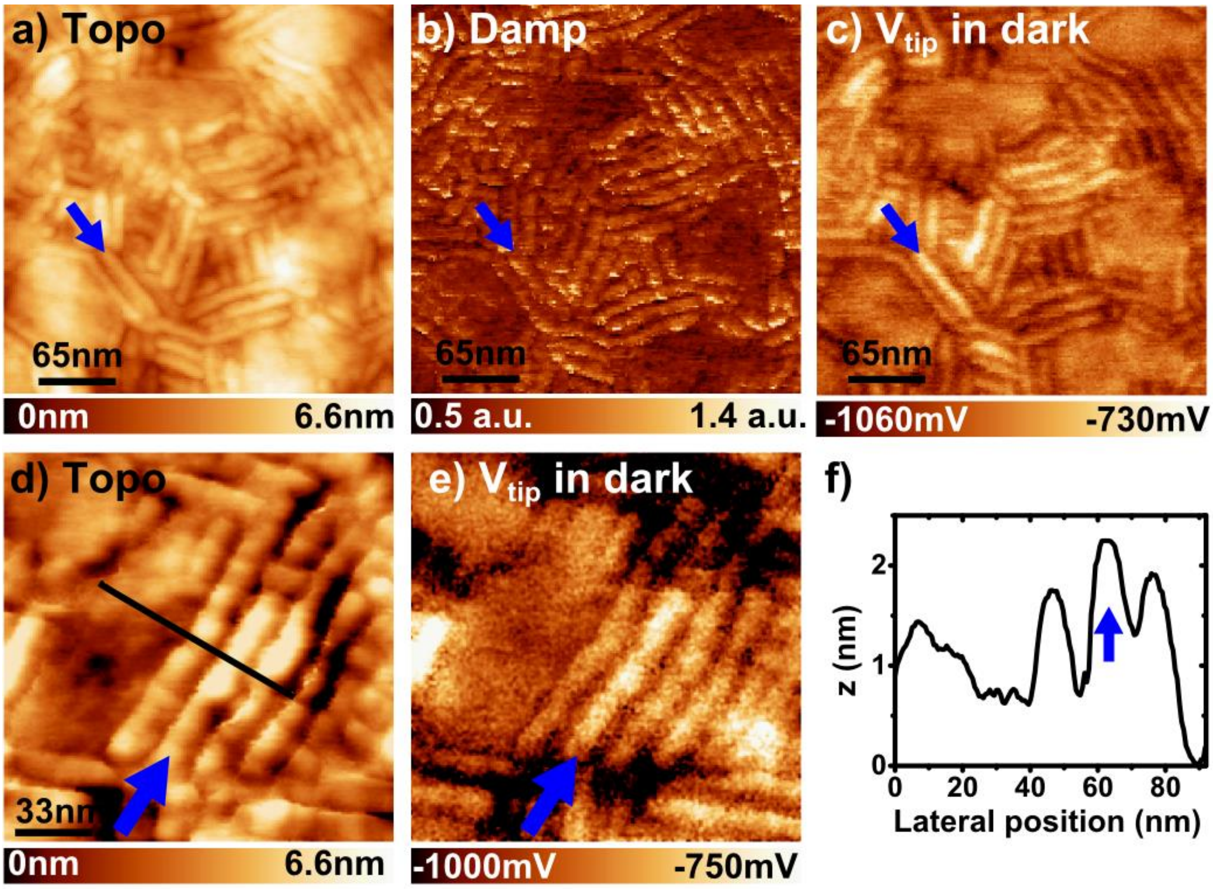

Figure S2: (a,b,c,) nc-AFM/KPFM images (325×325nm) of the AD3 film (Δf=-20Hz, A_{Vib}=20nm). (a) Topography (b) damping (c) KPFM potential recorded in dark. The blue arrow pinpoints a stack displaying a higher surface potential than its neighbors. (d,e) High resolution nc-AFM/KPFM images (165×165nm, Δf=-20Hz, A_{Vib}=20nm). (f) Topographic cross section corresponding to the path highlighted by a black line in (d).

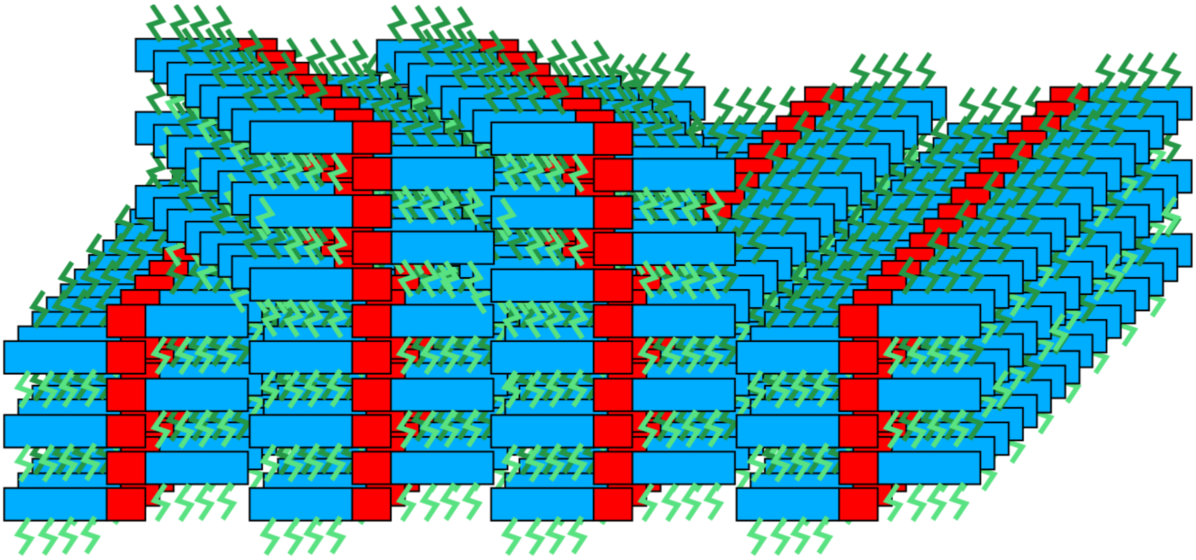

Figure S3: Schematic 3D representation of two lamellae with different in-plane π-stacking directions.

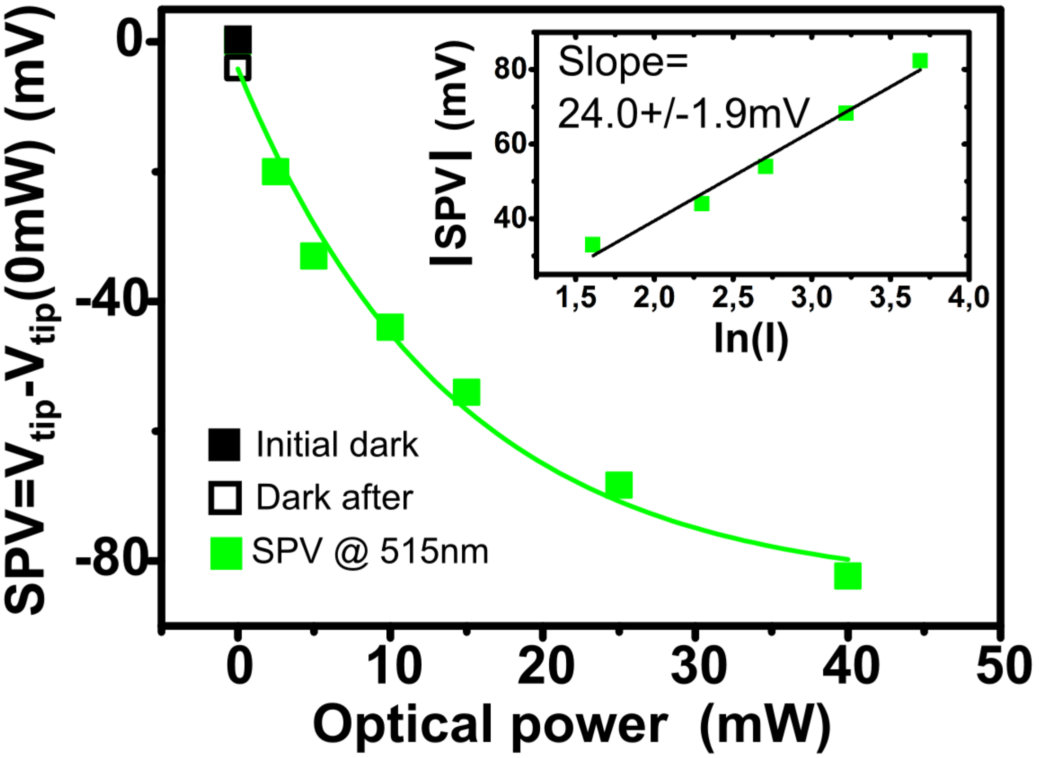

Figure S4: surface photovoltage (calculated as the difference between the tip compensation under illumination and the tip compensation in dark) as a function of the illumination intensity at 515nm. The in-dark potential values before and after the intensity sweep are represented by filled and open black squares, respectively. Inset: absolute value of the SPV as a function of the natural logarithm of the illumination intensity. The slope of the linear fit is equal (within the error bar) to k_BT/e=25mV, which indicates that the recombination is bimolecular.

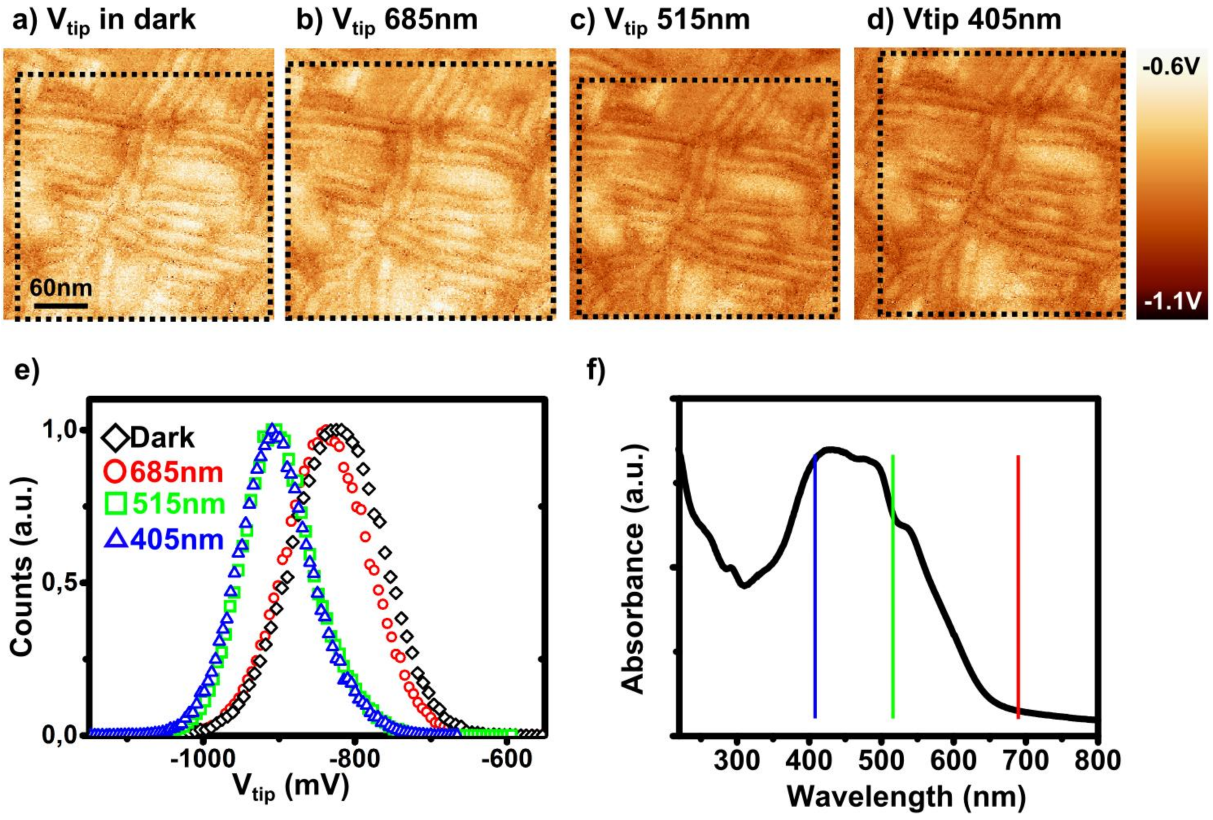

Figure S5: KPFM potential (tip compensation bias) images recorded approximatively over the same location, in dark (a), under illumination at 685nm (b), at 515nm (c) and at 405nm (d) (all wavelength with the same optical intensity). (e) Potential histograms extracted from the areas delimited by black dotted rectangles in (a,b,c,d,). There is almost no potential shift under illumination at 685nm, while the potential shifts are nearly identical for the data recorded at 515nm and 405nm. (f) This behaviour is consistent with the UV-visible spectrum for the AD3 dyad in the solid state. Moreover, the absence of potential shift at 685nm confirms the absence of any significant photovoltage related to the silicon cantilever itself.

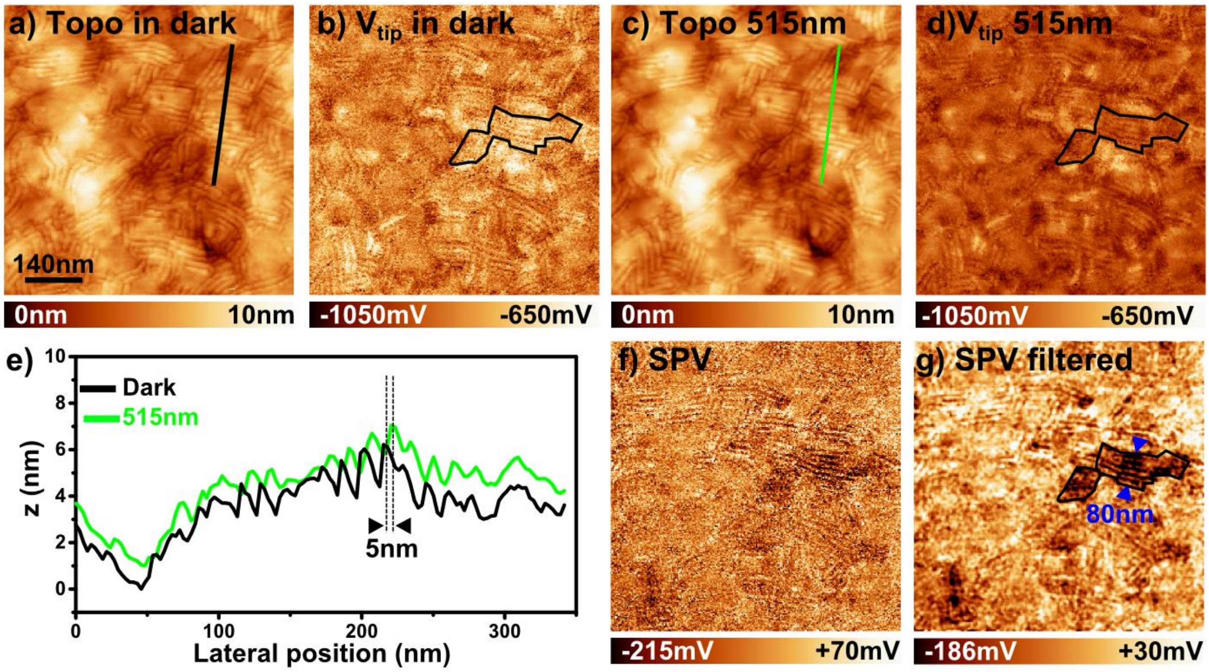

Figure S6: (a,b) Topographic and KPFM potential images (712×712nm) of the AD3 film (Δf=-10Hz, A_{Vib}=20nm) recorded in dark (c,d) Topographic and KPFM potential images recorded in the same area under illumination at 515nm. (e) Comparison between the topographic profiles simultaneously extracted from the topographic images in dark (black line) and under illumination (green line). (f) SPV image calculated as the difference between the KPFM images recorded under illumination and in dark). (g) SPV image filtered with a Gaussian smooth. The black contour highlights an area displaying a strongly negative SPV. Its lateral extension is much larger than the residual error that can be estimated from the topographic profile analysis.

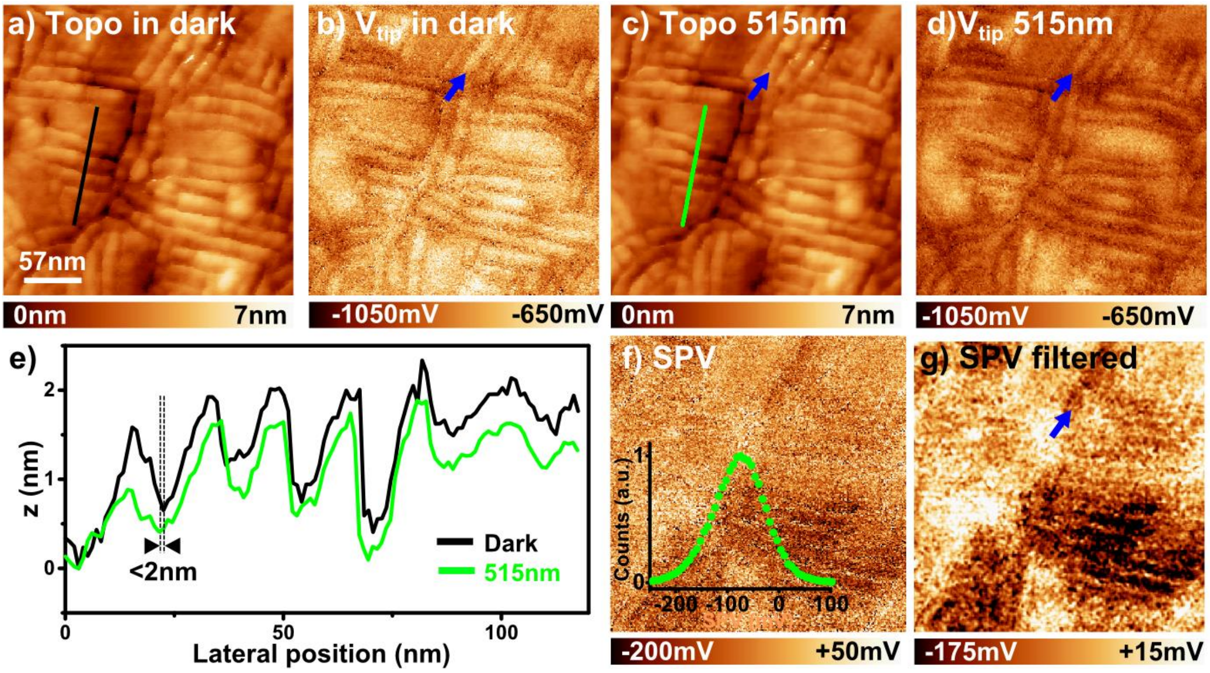

Figure S7: (a,b) Topographic and KPFM potential images (286×286nm) of the AD3 film (Δf=-10Hz, A_{Vib}=20nm) recorded in dark (c,d) Topographic and KPFM potential images recorded in the same area under illumination at 515nm. (e) Comparison between the topographic profiles simultaneously extracted from the topographic images in dark (black line) and under illumination (green line). (f) SPV image calculated as the difference between the KPFM images recorded under illumination and in dark). (g) SPV image filtered with a Gaussian smooth. The blue arrows pinpoint a supramolecular stack displaying a lower (*i.e*. more negative) SPV than its neighbours.

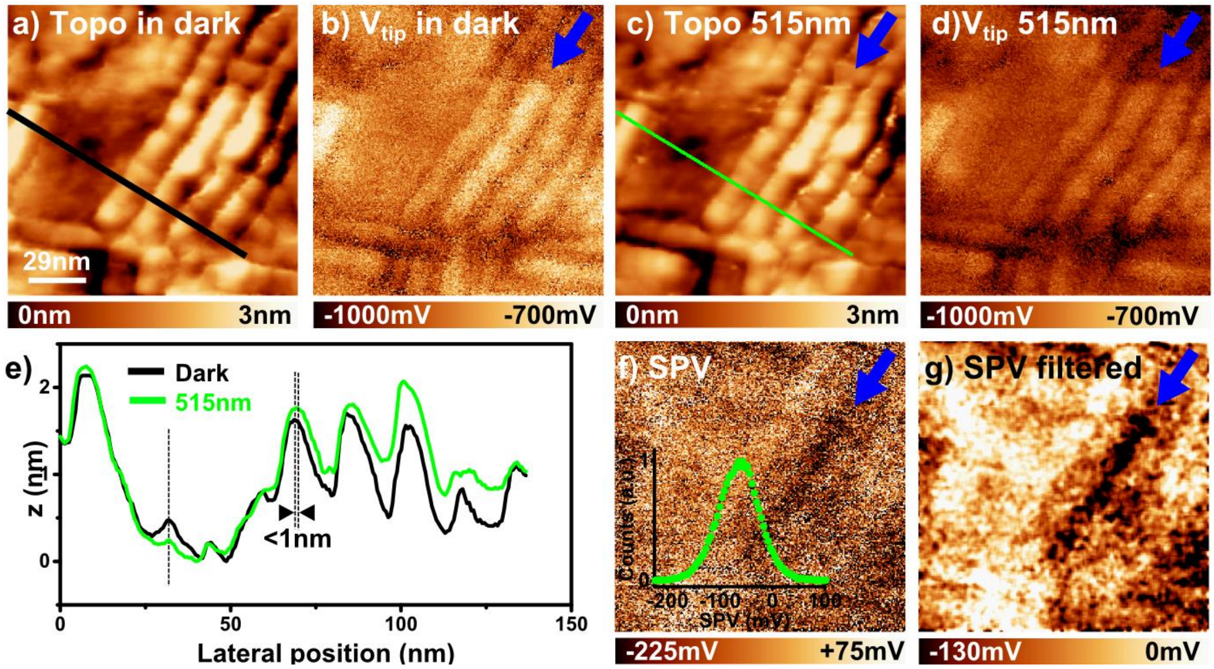

Figure S8: (a,b) Topographic and KPFM potential images (145×145nm) of the AD3 film (Δf=-10Hz, A_{Vib}=20nm) recorded in dark (c,d) Topographic and KPFM potential images recorded in the same area under illumination at 515nm. (e) Comparison between the topographic profiles simultaneously extracted from the topographic images in dark (black line) and under illumination (green line). (f) SPV image calculated as the difference between the KPFM images recorded under illumination and in dark). (g) SPV image filtered with a Gaussian smooth. The blue arrows pinpoint a supramolecular stack displaying a lower (*i.e*. more negative) SPV than its neighbours.

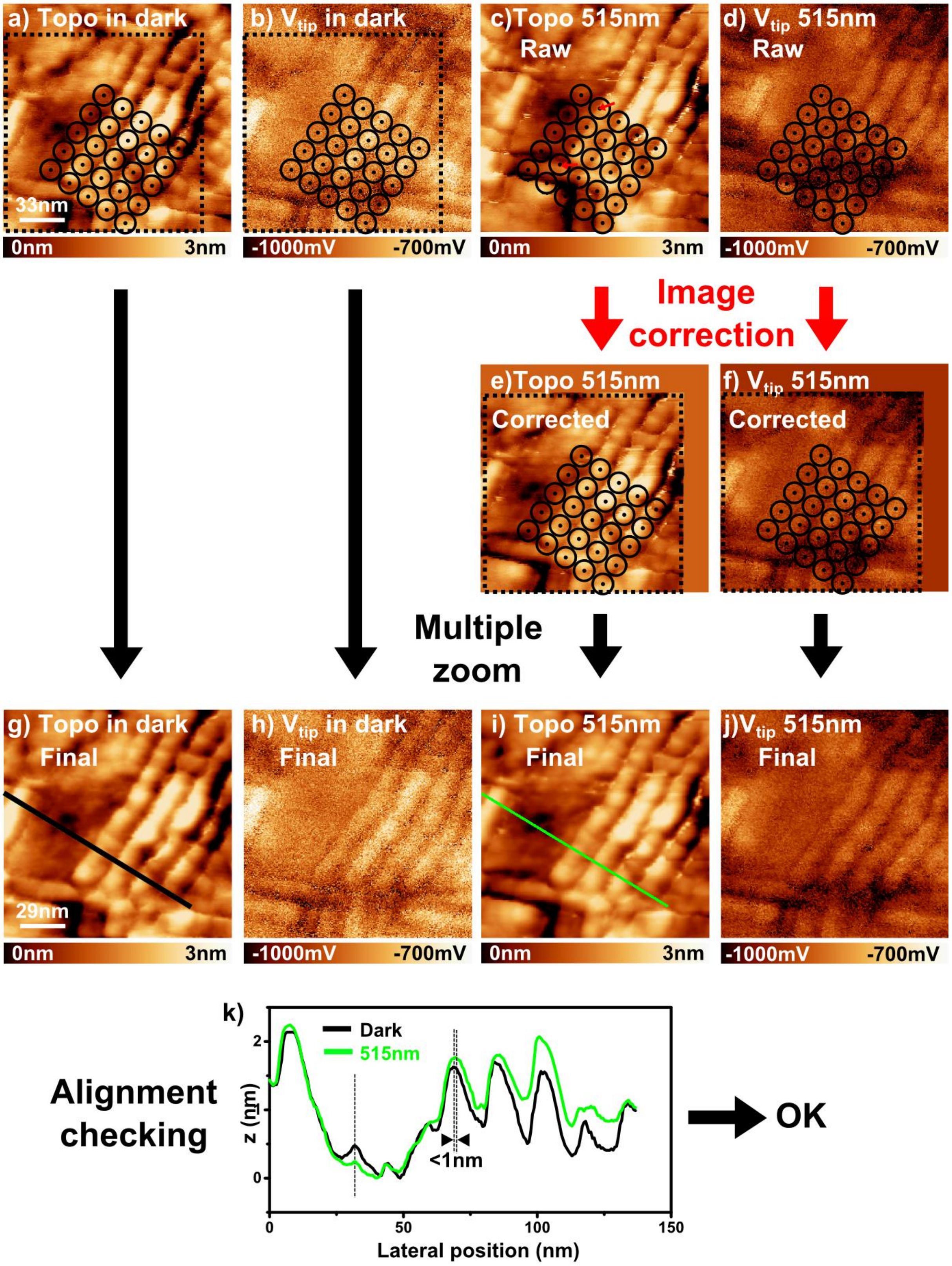

Figure S9: Correction procedure used for the SPV image calculation. (a,b,c,d) Set of 4 sources images. (a,b) Topographic and KPFM potential source images recorded in

dark. (c,d) Topographic and KPFM potential source images recorded under illumination. A lattice of reference points is defined in relation with the features displayed in image (a). This lattice is superimposed on images (c,d). An image correction (see the red vectors in (c)) is applied to achieve a similar matching of the topographic features with the reference lattice under illumination. The same correction parameters are simultaneously applied to the KPFM image. (e,f) Corrected topographic and KPFM potential images under illumination. Using a multiple zoom tool, a new set of images is extracted at the same locations from the in-dark images and the corrected images (under illumination). A final set of images is obtained (g,h,i,j) that can be used to calculate the SPV images. (k) The topographic profiles are used to check the quality of the image alignment after the correction procedure. This process is performed by using the lattice tool of the WSxM image processing software developed by I. Horcas *et al*. (Horcas, I.; Fernandez, R.; Gomez-Rodriguez, J.M.; Colchero, J.; Gomez-Herrero, J.; Baro, A.M. *Rev. Sci. Instrum.* **2007,** *78,* 013705).

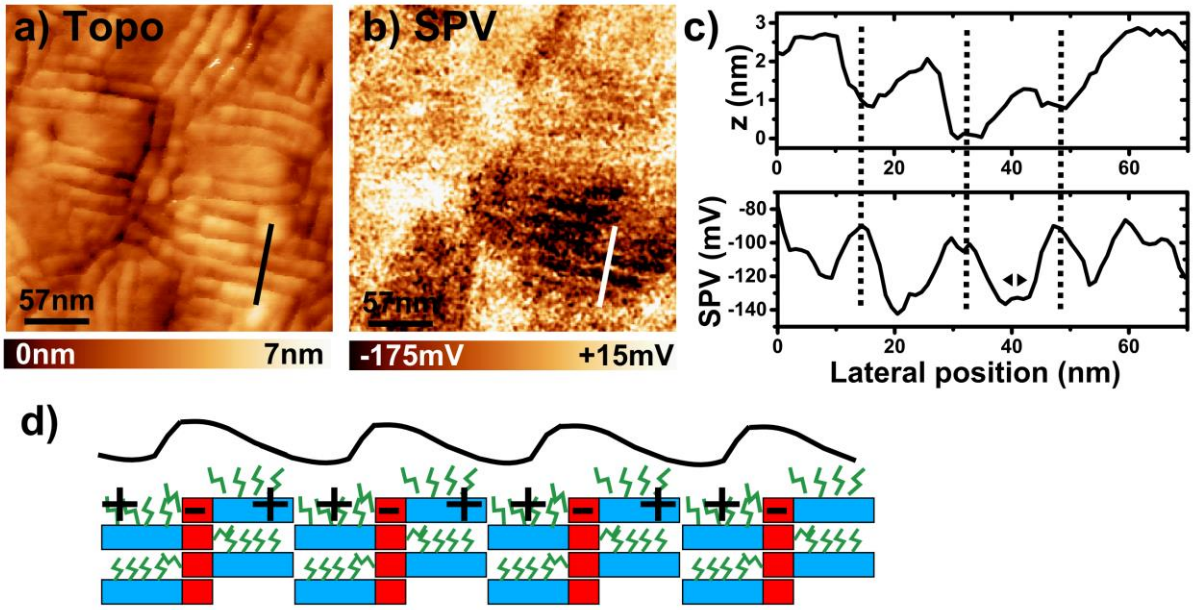

Figure S10: (a) 286×286nm nc-AFM topographic image of the AD3 film. (b) SPV image calculated as the difference between the KPFM images recorded under illumination and in dark. Lateral resolution: *ca.* 2nm. (c) Topographic and photovoltage profiles corresponding to the path highlighted in (a,b). (d) Schematic side view of the edge-on lamellae. According to this model, the local surface potential minima (over the negatively charged PDI blocks) shall approximatively correspond to the topographic maxima, and vice-versa.
